# Current Trends in the Optical Characterization of Two-Dimensional Carbon Nanomaterials

**DOI:** 10.3389/fchem.2019.00927

**Published:** 2020-01-28

**Authors:** Anton Kröner, Thomas Hirsch

**Affiliations:** Institute of Analytical Chemistry, Chemo- and Biosensors, University of Regensburg, Regensburg, Germany

**Keywords:** graphene, graphene oxide, carbon nanomaterial, optical characterization, methods, defects

## Abstract

Graphene and graphene-related materials have received great attention because of their outstanding properties like Young's modulus, chemical inertness, high electrical and thermal conductivity, or large mobility. To utilize two-dimensional (2D) materials in any practical application, an excellent characterization of the nanomaterials is needed as such dimensions, even small variations in size, or composition, are accompanied by drastic changes in the material properties. Simultaneously, it is sophisticated to perform characterizations at such small dimensions. This review highlights the wide range of different characterization methods for the 2D materials, mainly attributing carbon-based materials as they are by far the ones most often used today. The strengths as well as the limitations of the individual methods, ranging from light microscopy, scanning electron microscopy, transmission electron microscopy, scanning transmission electron microscopy, scanning tunneling microscopy (conductive), atomic force microscopy, scanning electrochemical microscopy, Raman spectroscopy, UV–vis, X-ray photoelectron spectroscopy, X-ray fluorescence spectroscopy, energy-dispersive X-ray spectroscopy, Auger electron spectroscopy, electron energy loss spectroscopy, X-ray diffraction, inductively coupled plasma atomic emission spectroscopy to dynamic light scattering, are discussed. By using these methods, the flake size and shape, the number of layers, the conductivity, the morphology, the number and type of defects, the chemical composition, and the colloidal properties of the 2D materials can be investigated.

## Introduction

Two-dimensional (2D) materials, especially graphene or graphene-related materials, have been studied extensively in the past decade regarding their outstanding properties such as mechanical strength, chemical inertness, high electrical and thermal conductivity, high mobility, or optical transmittance (Li et al., 2009; Allen et al., 2010; Zhu et al., 2010). These features lead to the assumption that graphene-related materials are promising candidates to be used in a large field of applications like high-power electrical or radiofrequency devices, batteries, and bio- and chemo-sensors or as membrane for water purification (Chen et al., 2012; Zhao et al., 2012; El-Kady and Kaner, 2013; Zhang et al., 2013).

Graphene was firstly cleaved off from graphite in 2004 by Geim and Novoselov via a scotch tape method (Novoselov et al., 2004). Since that time, a lot of progress was made, and many other layered materials have been exfoliated as two-dimensional nanomaterials (Zhang et al., 2018a). Researchers even report on the sophisticated hybrid materials, taking benefit, or creating new features by the functionalization or the combination of two or more nanomaterials. Individual 2D nanomaterials, such as graphene, BN, MoS_2_, and WS_2_, are already commercially available nowadays. Nevertheless, these products often suffer from little to no information on their exact properties such as size, number of layers, and defects.

The size of graphene ranges typically from several nanometers over micrometers up to millimeters, maintaining a thickness of only one atom at best. Bottom-up methods like chemical vapor deposition or epitaxial growth on SiC produce graphene in wafer-scale areas; top-down methods like chemical, mechanical, or electrochemical exfoliation generated graphene wherein the flakes have a very wide size distribution (Tang et al., 2012; Edwards and Coleman, 2013). Furthermore, the top-down methods produce graphene with different qualities in terms of the kind and the number of defects. These defects can have some benefits like improved dispersibility in water, or it can be detrimental since the electrical conductivity gets decreased (Araujo et al., 2012).

Furthermore, to tailor properties like chemical sensitivity, catalytic effects, or mechanical strength, such materials need to be modified by other nanomaterials and (bio-)molecules or by doping with other elements (Liu et al., 2012). Besides knowing the exact chemical composition of the 2D material, it is also of great importance to identify the contaminants or the impurities introduced during the fabrication, the modification, or the implementation into an application (Ambrosi et al., 2012; Chua et al., 2014). Moreover, to get a complete picture, more than one analytical method is often needed. Furthermore, the characterization often gets more difficult because the sample preparation for many techniques is not straightforward. In this review, the state-of-the-art characterization techniques of graphene-related materials in terms of flake size and shape, number of layers, morphology, number and type of defects, functionalization as well as colloidal properties are discussed.

## Characterization

Two-dimensional carbon nanomaterials need to be prepared, transferred, or modified in many different ways to get the benefit of their attractive features. Therefore, it is of great importance to characterize the material in each stage of synthesis or processing, but at the same time, characterization is extremely challenging due to the small dimensions and the needed accuracy as small variations in the shape, the dimension, or the composition of such materials can already greatly affect their properties. Several microscopic or spectroscopic methods for graphene characterization have been established and are reviewed in the following from the viewpoint of the material property which is desired to be investigated in detail.

### Size and Shape

**Figure 1:**
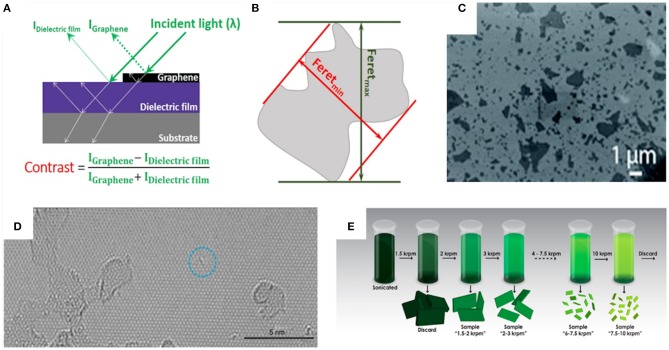
**(A)** A schematic illustration of the image generation of a layered graphene system using light microscopy due to optical transmission and reflection. **(B)** A schematic explanation of Feret_min_ and Feret_max_ diameters. **(C)** The SEM images of graphene oxide with high optical contrast and flake overlapping. **(D)** A TEM image of honeycomb carbon lattice next to lattice defects. **(E)** The centrifugation cascade of exfoliated graphene to reach a narrow graphene flake size distribution. **(A)** Adapted with permission from Tan et al. (2017). Copyright (2017) American Chemical Society. **(C)** Reproduced under a Creative Commons Attribution (CC BY 3.0) license from Gao (2017). **(D)** Reproduced under a Creative Commons Attribution (CC BY 4.0) license from Seiler et al. (2018). **(E)** Reprinted with permission from Backes et al. (2016b). Copyright (2016) American Chemical Society.

A typical feature of a nanomaterial is the change of its properties with size and shape. The same is true for the 2D materials. These materials intrinsically do not have any bulk phase, which means that every single atom is a surface atom. Nevertheless, at the edges, the valences of such atoms might be different compared to those located within the flake. The chemical and physical properties of the graphene flakes get affected by the degree of sp^2^-conjugated carbon atoms, and thereby it is important to measure the size and the shape of the 2D materials. The size of spherical objects (0D material), like monodispersed nanoparticles, can be fully described by the radius and is therefore easy to be characterized. In contrast, non-spherical nanomaterials, such as all kinds of 2D materials, demand a more complex characterization. Exfoliation-based preparation methods lead to irregular shapes and a large distribution in sizes. Therefore, at least the average length and width have to be known to get a first impression of the flake size and of the surface area. Top-down syntheses produce graphene flakes ranging from several micrometers down to a few nanometers in size. A detailed statistical analysis of the flake size distribution is recommended (Modena et al., 2019) as the mean values together with the standard deviations are only useful for a Gaussian distribution of the flake sizes. If two or more populations are predominantly present, a median value, representing 50% of the population which is below or above, or a mode size ascribed to the fraction with the highest frequency will be more informative. The 2D materials can also exhibit holes within a single flake, which makes the characterization even more sophisticated. This might be important for the design and the characterization of graphene membranes for separation or in electronic applications (Banhart et al., 2011; Feicht and Eigler, 2018). An exact knowledge of the size and the shape is especially necessary when changes in dimensions tremendously impact the physical properties, such as electronic transport, absorbance, or luminescence (Khan et al., 2012; Chen et al., 2015; Coleman et al., 2017; Zhao et al., 2017). The Feret diameter is a valuable parameter for the characterization of flake sizes as its value considers the irregularities in shape (Walton, 1948). To obtain the Feret diameter, the area of an individual flake has to be fitted by two tangents which are parallel to each other. The maximum distance between both tangents is assigned as the Feret diameter or Feret_max_. Since the Feret diameter includes precise information in only one dimension of a graphene flake, it is necessary to measure a second Feret diameter, the so-called Feret_min_ diameter, which measures the minimum distance between the two tangents, rotated by an arbitrary angle (Figure 1A). Both values can be combined to a single value, referring either to the ratio (Feret_ratio_) or to the average (Feret_mean_) (Walter et al., 2015).

To determine the flake size distributions, one needs to image a large number of individual flakes with high resolution to be able to get a reliable statistical analysis. Scanning electron microscopy (SEM) or high-resolution transmission electron microscopy (HRTEM) are suitable, but for large flake sizes excessing the nanometer range, they might run into limitations caused by the slow throughput or by their resolution (Liscio et al., 2017). Surprisingly, it is also possible to observe single graphene flakes by light microscopy (LM) due to the reflections and the interferences of the incident light at the flake surface and at a substrate of choice, when an optical transparent layer of a certain thickness separates both. The flake gets depicted by its optical contrast, which depends mainly on the differences in the refractive index and the respective absorption coefficients between the observed material and the substrate (Figure 1B). The commonly used substrates for graphene are silicon wafer coated by a silicon oxide layer with a thickness of around 90 nm (Bayle et al., 2018) or 300 nm (Blake et al., 2007) under white light illumination. Even better contrasts can be obtained by using an excitation wavelength of 543 nm and a silicon nitride layer of 72 nm on the silicon substrates (Jung et al., 2007). Graphene flakes up to a few micrometers in size can be screened very fast and precisely by this method. With a suitable software, e.g., the open source project imageJ, such high-contrast pictures can be automatically analyzed by transferring the images into 8-bit monochrome images and by performing a Gaussian fit (Lin et al., 2012; Chakraborty et al., 2016). Whenever possible, it is recommended to perform an automated analysis as those are not biased by any expectations of the user. The analyses of microscopic images are often challenged by the fact that the flakes overlap each other. This makes an accurate evaluation of the size nearly impossible. When the flakes can be dispersed, the issue of overlapping can be overcome by dilution of the sample before applying it to the substrate. Nevertheless, the sample preparation can become a tedious work.

If the graphene flakes are in the nanoscale, SEM is the method of choice (Chen et al., 2016). To gain a high resolution, the type of substrate is essential. As for LM, the image quality of SEM pictures depends on the substrate. Here copper (Çelik et al., 2016) or silicon oxide (Grimm et al., 2016) performs very well (Figure 1C). Moreover, for a successful visualization of graphene by using SEM, good electrical conductivity between the sample and the sample holder must be ensured to prevent the charging effects by the e-beam (Kim et al., 2010). To achieve an atomic resolution or to detect vacancies in the atomic framework, HRTEM has been successfully applied (Seiler et al., 2018). By the same method, the defects in the carbon lattice of GO with only a few-atom resolution have been successfully visualized (Figure 1D) (Feicht and Eigler, 2018). With liquid cascade centrifugation, it was proposed that the mechanically exfoliated 2D nanomaterials (WS_2_ and MoS_2_) can be discriminated by their flake size. This was proven by the characterization of the flake size distribution by transmission electron microscopy (TEM) and atomic force microscopy (AFM). From these analyses, metrics have been developed to characterize the size from the extinction spectra of the dispersion containing the respective nanomaterials (Figure 1E) (Backes et al., 2016a).

A perfect graphene flake consists exclusively of six-membered carbon rings only; as a consequence, only the angles exactly 60° and 120° will characterize the graphene flake borders. From theoretical modeling, it is known that the zigzag graphene edges have specific magnetic properties which are interesting for spintronic devices (Wang et al., 2008). The triangular graphene flakes should theoretically consist of zigzag edges only, and by this, it is desired for certain applications to create flakes of this shape (Ci et al., 2009). This goes along with the need of a characterization method to investigate the structure of the carbon atoms forming the border. The shape of the graphene flakes can be easily influenced during synthesis by the CVD processes that involve changing the growth parameters like temperature, methane flow rate, or growth direction and finally confirming by SEM and AFM measurements (Fan et al., 2011).

### Number of Layers

**Figure 2:**
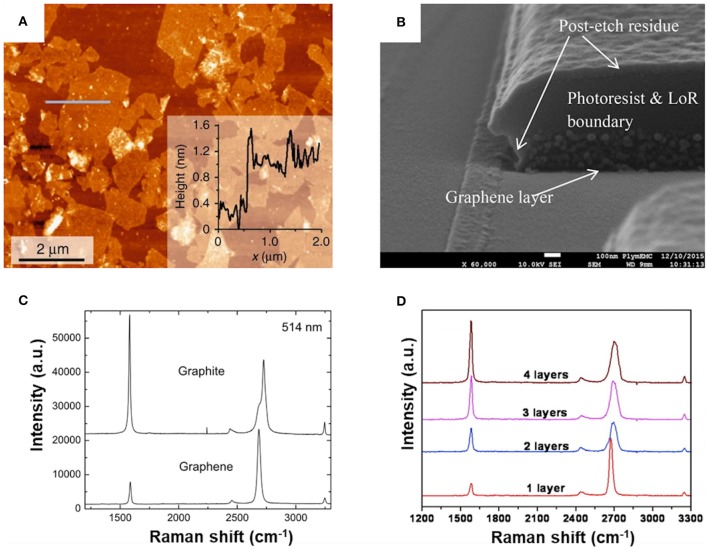
**(A)** An AFM image of exfoliated graphene with corresponding height profile along the *gray line*. **(B)** A SEM image of a focused ion beam cross-section through a graphene layer. **(C)** The Raman spectra of graphite and graphene at 514 nm. **(D)** The Raman spectra of graphene with a different number of layers at 532 nm. **(A)** Reproduced under a Creative Commons Attribution (CC BY 4.0) license from Seiler et al. (2018). **(B)** Reproduced under a Creative Commons Attribution (CC BY 4.0) license from Li et al. (2017). **(C)** Reprinted with permission from Ferrari et al. (2006). Copyright (2006) American Physical Society. (D) Reprinted and adapted from Wang et al. (2008). Copyright (2008) American Chemical Society.

Per definition, graphene consists of a single carbon layer only (Kochmann et al., 2012). Nevertheless, individual graphene flakes can be arranged in stacks to create the special properties or as an unwanted byproduct obtained by top-down fabrication methods. Few-layer graphene can also arise upon agglomeration when stored in dispersions or after assembly from the liquids on a substrate. To determine the thickness of the graphene layers or the number of layers of stacked graphene flakes, several methods can be used. Here similar requirements have to be fulfilled to determine the number of layers for the characterization of the flake size. For example, the optical contrast between the graphene and the substrate must be sufficiently high to resolve few-layer graphene by optical or electron microscopy. The number of graphene layers on the substrate can be estimated by correlation between the contrast and the specific thickness: e.g., the number of graphene flakes has been determined by using 7.5 nm Au, 1 nm Ti, and 93 nm SiO_2_ stacked on a silicon wafer, and by analyzing the reflected light collecting the image after a 520-nm band pass filter by its optical contrast (Velický et al., 2018). A disadvantage of this method is that the graphene layer thickness can be estimated only by knowing the thickness of one single layer of the 2D nanomaterial. Since the layer thickness of many carbon 2D materials is affected by the number of defects and therefore depends on the degree of oxidation, an even more precise method, such as AFM, can be applied (Figure 2A). To reach resolutions in height smaller than 1 nm over a large lateral area, it is absolutely necessary to use substrates like mica or large salt crystals which are known to be atomically smooth. Again, the transfer of the material onto the substrate without inducing wrinkles or depositing impurities is challenging. Also, flake overlapping needs to be avoided. The differences in thickness of <1 nm have been resolved (Khan et al., 2017; Halbig et al., 2018), and also the number of the graphene layers (from one layer up to 10 layers) was determined (Pu et al., 2009; Tan et al., 2017; Velický et al., 2018). To characterize the thicknesses of the deposited graphene layers, focused ion beam (FIB) cuts can be fabricated and analyzed by electron microscopy. Here the sample is bombarded by ions or electrons which generate a cut into the graphene layer. Subsequently, a SEM image of the cross-section reveals the layer thickness of the deposited graphene (Figure 2B) (Schnitker et al., 2018). The FIB cut method comes with the disadvantage of destroying the sample. In addition, the sample preparation and the measuring time for FIB cuts are higher compared to those for standard SEM images. The non-conductive samples have to be taped or sputtered with metals like silver, gold, or platinum to prevent electrical (over)charging in the SEM microscope. The typical thicknesses of the metallic overlayer are 10 nm and therefore usually much thicker than the 2D material itself. This means that the sputtering can have a big impact on the 2D material layer thickness, especially if one is interested in the thickness of the loosely stacked graphene flakes as they might be used in membranes, which can be easily compressed by the metallic layer deposited on top. Furthermore, it is challenging to derive a homogeneous thickness of the sputtered material in the *x*- and *y*-direction because layers that are too thin lack in conductivity and still lead to overcharging, whereas layers that are too thick obscure the fine details and make reliable thickness determination nearly impossible (Golding et al., 2016). Whereas, FIB cut is not ideal to visualize the monolayers of graphene, the strength of this method is the thickness determination of the coated, the dispensed, or the printed graphene layers. Graphene thickness can also be determined by the optical contrast of TEM. Ghosh et al. checked the number of graphene layers by HRTEM in energy storage applications (Ghosh et al., 2018). Since a substrate is necessary for the printed or the dispensed graphene by using inks, TEM is not applicable for this kind of characterization.

In addition to the imaging methods, the layer thickness and the number of layers, respectively, can also be determined by Raman spectroscopy. With an increased number of graphene layers, the G-peak is subject to a slight shift to lower wavenumbers, whereas the D-peak undergoes changes in shape, width, and position (Figures 2C,D) (Ferrari et al., 2006). The intensity ratio of the maximum of the 2D-band to the G-band can be used as a possible quantitative metric for the determination of the number of graphene layers (Backes et al., 2016a). An empirical model revealed the following equation to calculate the number of graphene layers (< *N* >):

$$\begin{array}{l} <N>\;=1.04\;{\frac{I_{2D}}{I_{G}}}^{-2.32} \end{array}$$

It is still questionable if such an empirical formula is valid for all combinations of materials, e.g., it needs to be validated if the coefficients in this model need to be adapted by experimental settings, e.g., by the laser wavelength of the Raman spectrometer. Nevertheless, this method has the advantage to estimate the number of graphene layers in a fast and cheap way and over a large sample area. The disadvantages of this method come with the fact that the error of this metric is around 25% (Backes et al., 2016a), and therefore only rough estimations are possible, which might be useful in a process control during a fabrication step where high throughput is important. For such purposes, it would also be attractive to refine the metrics according to the given materials and equipment.

### Morphology

**Figure 3:**
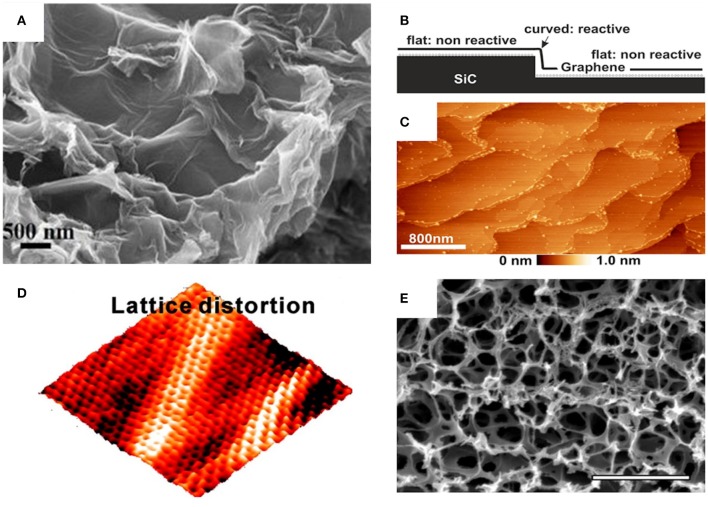
**(A)** A SEM image of rGO with highly wrinkled morphology. **(B)** A measuring setup to characterize the large graphene terraces at ambient conditions. **(C)** An AFM image of graphene. The high spots indicate reactive edges. **(D)** A 3D STM image of graphene which demonstrates the clear lattice distortions. **(E)** A SEM image of nano-porous graphene for the determination of pore size and pore size distribution. **(A)** Reproduced under a Creative Commons Attribution (CC BY-NC-ND 3.0) license from Chabot et al. (2013). **(B,C)** Reprinted with permission from Wastl et al. (2014). Copyright (2014) American Chemical Society. **(D)** Reprinted with permission from Zhang et al. (2011). Copyright (2011) American Chemical Society. **(E)** Reproduced and adapted under a Creative Commons Attribution (CC BY 4.0) license from Lu et al. (2016).

The surface roughness or topography significantly contributes to the graphene flake surface area and is mainly introduced by the defects in the carbon lattice. It is especially of interest in any application taking benefit of a large surface area or special surface properties, e.g., binding of absorbents in chemical sensing (Pumera et al., 2010) or as a catalyst in synthesis applications (Rodríguez-Reinoso, 1998). On the one hand, defects, such as those due to epoxy or hydroxyl groups, distort the atomically smooth surface of graphene, and on the other hand, defects caused by the distortion on the honeycomb lattice, such as five- or seven-membered rings as well as carbon atoms exchanged by nitrogen and boron, affect the topography of the material as described by the wrinkles in the material. The surface roughness of individual graphene flakes is typically in the low-nanometer range and can be characterized by AFM, scanning tunneling microscopy (STM), or SEM. When using SEM, low-acceleration voltages in the range of 3 kV (Joy and Joy, 1996) need to be used to keep the penetration depth of the electrons low so as to enable highest surface sensitivity. Since the electrons have a small reach in matter, SEM images made by secondary electrons are extremely surface-sensitive and can resolve the surface morphology (Figure 3A). To get information on the surface morphology in atomic resolution, AFM is the method of choice. The resolution of AFM—typically in the 1-nm range—is sufficient to resemble even absolute dimensions and therefore allows an easier comparison to each other. Investigations of few-layer graphene at different temperatures showed that the graphene roughness can be influenced by temperature (Zhou et al., 2018). Moreover, by using AFM, an influence on the surface smoothness by the degree of oxidation of the carbon nanomaterial was demonstrated. Exposure to hydrogen iodide vapor smoothens the surface by reducing the graphene oxide. This indicates the successful elimination of oxygenated functional groups of graphene oxide (Fakharan et al., 2019). An impressive AFM study on the hydrogen-intercalated epitaxially grown graphene on SiC (0001) demonstrates that curvatures or steps in the graphene are favored spots for adsorbates. The authors achieved, for the first time, an outstanding resolution of 0.3–0.4 nm at ambient conditions (Figures 3B,C) (Wastl et al., 2014). In contrast to this method, the roughness of a graphene surface was also resembled in atomic resolution by using STM (Figure 3D). The morphology differences even in the very low angstrom range (< 0.5 Å) can be resolved (Järvinen et al., 2013), but this method suffers from the need of vacuum conditions for measurement.

Besides the single graphene flakes, graphene films consisting of many flakes are also of interest in terms of the characterization of their topology. For screening of the homogeneity of the graphene films, optical microscopy images can be used. Wrinkles, overlapping flakes, and non-confluently assembled graphene films deposited from graphene suspensions can be investigated in a simple manner (Lian et al., 2018). Inhomogeneous graphene layers with the so-called coffee ring characteristics, which are typical for dispensing and drop-casting of nanomaterials, often needs to be avoided since the properties, e.g., layer thickness or electrical conductivity, are different at the coffee ring compared to the rest of the graphene layer (Gorkina et al., 2016). By observing a Raman line scan over the deposited graphene area or layer and calculating the *I*_D_/*I*_G_ ratio, it is possible to obtain information about the oxidation degree and the location of defects and therefore the homogeneity of the dispensed or the printed graphene layers (Dresselhaus et al., 2010).

Moreover, porous materials, such as graphene aerogels, are characterized by a rough surface and an uneven topology. Typical for this class of materials is the large surface area, which can be used for the intercalation or the absorption of gases, liquids, metals, or ions. Especially parameters like the pore size, which is typically in the range of a few nanometers, and the pore size distribution are of great interest for applications like absorption membranes, energy storage components, or nanoelectronics (Cohen-Tanugi and Grossman, 2012; Russo et al., 2013). For the investigation of the pore diameter as well as the pore distribution, SEM (Figure 3E) has been preferably used (Yang et al., 2016). Furthermore, the pore density as well as the channels formed by pores can be studied (Yousefi et al., 2019). In another example, graphene oxide was reduced, and porosity was introduced by a NaOH treatment followed up by an acid treatment to ensure that the carboxylic functionalities remain acidic. Pores with an average diameter of 2.16 nm and a pore density of 5.74% have been obtained and characterized by the pore area analysis of STM images (Su et al., 2015). For assemblies of 2D materials consisting of channels that form fluidic networks, as desired for filtering applications, detailed information about the pore size is indispensable. It was shown by AFM that the average pore size can be tailored from 3.7 nm upon 6 h of γ-ray irradiation up to 13.6 nm for a 24-h treatment (Yu et al., 2016). While AFM and STM, with their convincing resolution in the nanometer to the sub-nanometer range, are therefore superior to SEM, both methods suffer in terms of applicability and throughput.

Microscopy techniques of all kinds mainly characterize the outer receptively visible surface of the porous materials; a further method where the total surface area, meaning the inner and the outer surface, can be investigated has to be introduced. For that, Brunauer–Emmett–Teller (BET) studies are helpful (Osaki, 2018). Here typically N_2_ is introduced into a sample chamber and is adsorbed on the material to be investigated. Finally, the adsorbed gas is measured, and the entire surface can be calculated. Microporous graphene paper can be used as the air cathode for the Li–O_2_ batteries fabricated, and the total surface area (around 373 m^2^ g^−1^) was determined by BET adsorption (Kim et al., 2016). Furthermore, by using BET, the influence of graphene oxide reduction on the total surface and on the average pore width can be characterized and determined. BET showed that the reduction method via ascorbic acid leads to more surface area and a smaller average in pore width compared to the reduction with urea (The Vinh et al., 2019).

### Chemical Functionalities

Fabrication methods for the 2D materials following a top-down approach are additionally needed to identify and to characterize contaminations, impurities, and functionalities introduced during the synthesis. The importance arises from the fact that even low contaminants bear the risk of changing the chemical and physical properties of the nanomaterial. The comparison of differently prepared materials is not an easy task up to now as there are no widely accepted standards in material characterization that have been established, e.g., in organic chemistry, by giving data from nuclear magnetic resonance and mass spectrometry (MS) when a new compound is reported. During fabrication, the carbon nanomaterials get contaminated by the species used for the preparation, the purification, or the transfer process (Smith et al., 2014). Especially chemicals with high binding affinity to the large surface of carbon atoms need to be determined.

A widely used method to fabricate graphene is the chemical or electrochemical oxidation of graphite (Shams et al., 2015). These methods introduce the different oxygen functionalities like hydroxides, epoxides, and carbonyl or carboxyl groups into the honeycomb graphene lattice. By changing the parameters in the synthesis, the number and kind of such groups can be influenced, and therefore the degree of functionalization allows the tuning of many properties such as electrical conductivity or dispersibility of the 2D materials.

**Figure 4:**
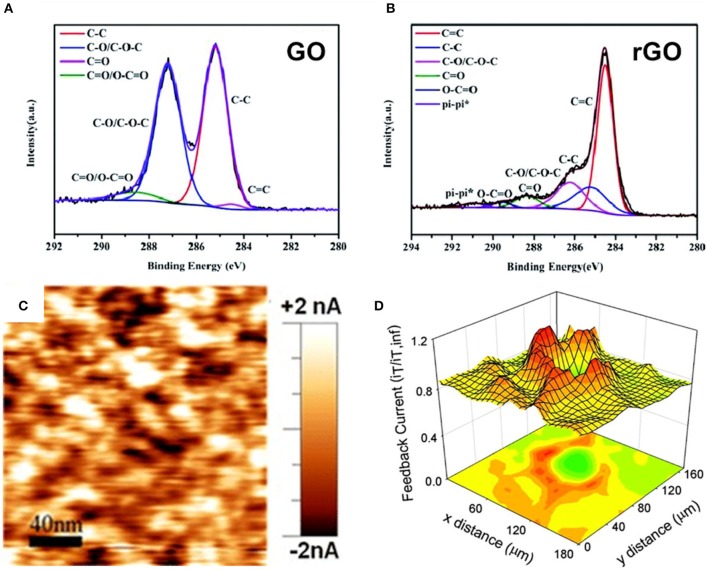
**(A)** An XPS C1s spectrum of GO with the corresponding C1s spectrum of rGO **(B)**. **(C)** A CAFM map of graphene with clear current discontinuity (the *darker spots* correspond to a lower conductivity). **(D)** A SECM 3D image of monolayer graphene with higher feedback current around the defect/edges. **(A,B)** Reproduced under a Creative Commons Attribution (CC BY-NC 3.0) license from Shen et al. (2018). **(C)** Reprinted with permission from Eckmann et al. (2012). Copyright (2012) American Chemical Society. **(D)** Reprinted with permission from Tan et al. (2012). Copyright (2012) American Chemical Society.

The characterization of the chemical composition qualitatively as well as quantitatively is routinely performed by X-ray photoelectron spectroscopy (XPS). A huge advantage of this method comprises high surface sensitivity. The nature of functionalities can be retrieved from the characteristic binding energy of every element, e.g., the C1s peak of carbon (at about 286 eV) and the O1s peak (at around 532 eV) are present in the XPS spectra of graphene prepared by exfoliation techniques. A shift in the binding energies is attributed to the binding partners of every atom, and therefore it is possible to determine the exact moiety of an oxygen functionality (Stobinski et al., 2014) (Figures 4A,B). XPS has also been applied to determine the oxidation degree of GO (Krishnamoorthy et al., 2013; Thebo et al., 2018). Another method for chemical characterization is electron energy loss spectroscopy (EELS). Here electrons interact with the sample by inelastic scattering, which results in the loss of energy (Egerton et al., 2004). By observing the C and O k-edge peaks, which represent the respective 2p partial density of states above the Fermi level, it is possible to investigate the degree of oxidation of graphene (Mkhoyan et al., 2009; Bellunato et al., 2016). Since EELS is often implemented as an additional feature of HRTEM, it is popular to generate the EELS spectra with atomic resolution, which is superior to XPS (Egerton, 2009). For large areas or when the lateral resolution is not of such importance, the conductivity changes in graphene can be measured by conductive atomic force microscopy (CAFM) (Figure 4C). Here a small tip, which also deals as an electrode, scans over the sample surface. It is possible to generate the conductivity maps of the graphene flakes or layers (Iwan et al., 2018; Putri et al., 2018). The CAFM proved that the inhomogeneities of a substrate surface have an influence on the graphene conductivity (Giannazzo et al., 2018) and that the conductivity within a graphene flake is also influenced by domains and wrinkles (Ahmad et al., 2011).

A very similar technique to characterize the surfaces or the surface interface is scanning electrochemical microscopy (SECM). SECM is based on the spatially resolved imaging of electrochemical processes that is detected by micro- or nanoelectrodes. If the applied voltage is sufficiently high, Fe^3+^ is reduced to Fe^2+^ at the electrode tip and generates a diffusion-controlled current, the so-called Faraday current. By measuring the Faraday current, information about the electrochemical reactivity and therefore about the defects in the graphene lattice can be obtained (Figure 4D) (Tan et al., 2012). The electro-activity of the reduced-graphene-oxide-coated polyester fabrics was investigated by SECM. It was shown that an increase in the electro-activity can be observed after the reduction of graphene oxide to reduced graphene oxide. Since the measured current depends on the distance between the tip and the sample, it is possible to get information about the surface topology when the tip is held at a constant height. SECM maps visualized the surface morphology of the reduced-graphene-oxide-coated fabrics (Molina et al., 2013). These examples show that SECM is an important technique to characterize the surface or the surface properties of graphene and other 2D materials. SECM is a valuable characterization method especially for the 2D catalysts where the surface reactivity can be locally monitored. SECM is still limited in its resolution as it is challenging to fabricate ultramicroelectrodes (Bard et al., 1990).

The oxygen-containing functionalities in carbon 2D materials can also be investigated by X-ray diffraction (XRD), wherein the (0 0 2) diffraction peak at around 25°-30° indicates the distance between the graphene layers and the (1 0) diffraction peak at around 40°-45° indicates the short-range order in the stacked graphene layers (Stobinski et al., 2014). By using XRD, it was possible to monitor the different degrees of oxidation when synthesizing GO by four different chemical exfoliation methods, as indicated by the change in the GO layer distance (Rodriguez-Pastor et al., 2015). Furthermore, the graphene layer distance is correlated with the degree of oxidation since the oxygenated functionalities are located perpendicular to the basal graphene plain (Drewniak et al., 2016). Also, thermal treatment affects the structure of the reduced graphene oxide, which was followed by comparing these with the (0 0 2) diffraction peak. A shift from 4.79° for the graphene oxide to 11.92°C for the reduced graphene oxide was found after a furnace process at 2,000°C under argon atmosphere (Song et al., 2013). XRD is also a powerful characterization method when the successful fabrication of heterostructures needs to be proven, as demonstrated for an electrocatalytic application where the graphene oxide was modified with Pd/Ni nanoparticles (Revathy et al., 2018). For that, the XRD plots of the graphene oxide, the Pt/Ni alloy, and the final graphene composite material were compared, and all the peaks of the composite were assigned to the starting materials. By matching all the peaks of the composite material to the corresponding starting materials, it is possible to successfully confirm graphene modifications.

Energy-dispersive X-ray spectroscopy (EDX) or X-ray fluorescence analysis (XRF) measures the characteristic X-ray radiation of every element in the sample. Whereas, EDX is always applied in combination with any kind of electron microscopy and therefore irradiates the sample with electrons, XRF uses X-rays. By introducing heteroatoms into the graphene lattice, the electronic, mechanical, or chemical properties can be tailored. EDX measurements confirmed the successful introduction of germanium into the graphene lattice for a later application in catalysis (Tripathi et al., 2018). For another catalytic application, Cr^6+^ should be reduced to Cr^3+^ by graphene, and therefore the material was modified by 3-aminopropyltriethoxysilane-stabilized Pt nanoparticles. The presence of platinum and silicon in the corresponding EDX spectrum confirmed the successful functionalization of graphene (Celebi et al., 2017). Also, the sulfur contaminations in the high ppm range on the reduced graphene oxide sheets, introduced by the use of sulfuric acid during the fabrication process, have been identified by EDX (Alam et al., 2017). Furthermore, the location or distribution of heteroatoms can be revealed by analyzing an EDX map (Pendashteh et al., 2017). Compared to EDX, the XRF measuring setup is less complicated, and the detection limit of XRF is lower (XRF ppm; EDX 0.1%) (Jembrih-Simbürger et al., 2002; Tiwari et al., 2005). Additionally, no vacuum is needed because no scattering takes place between the X-rays and the air. Also, liquid samples like graphene suspensions or inks (in glass tubes) can be characterized by XRF (Melquiades and Appoloni, 2004). The contamination introduced during graphene synthesis can be determined by XRF very easily (Jankovský et al., 2016).

One feature where XRF and EDX differ from XPS and AES is the penetration depth (Linke and Schreiner, 2000). In the case of XPS, the collected electrons result from the outer photoelectrical effect (Venezia, 2003), and for AES they are originated by the Auger effect (Reniers and Tewell, 2005). Both methods are comprised by an extremely low penetration depth of a few nanometers (few atom layers), allowing to get information on the chemical composition of the surfaces (Chang, 1971; Baer and Engelhard, 2010). Compared to XRF, both methods suffer from operation in high vacuum, which complicates the device setup and the sample preparation. Moreover, the probability of Auger effect is decreasing with increasing atomic number. Due to the competition to X-ray transition, quantitative detection is practically limited to the lighter elements (Chang, 1971). AES is limited to the conductive samples. Nevertheless, it is a powerful technique to investigate mainly the contaminations on the surfaces or thin layers (Powell et al., 1999), e.g., iron impurities introduced by the transfer of graphene from nickel to Si/SiO_2_ substrates were identified as contaminants caused by using FeCl_3_ to etch the Ni substrate (Xu et al., 2010). The graphene modifications of AgBr@Ag/N rGO and the chemical composition of the nitrogen-doped graphene composite have been identified with AES. Due to the presence of Br^−^, Ag^+^, and Ag^0^, they came to a result wherein some Ag^0^ are coated by AgBr (Zhang et al., 2018b). Also, the successful linking of the hexagonal boron nitride and GO was confirmed by AES (Bhimanapati et al., 2016). To characterize the non-conductive samples or to get more detailed information on the graphene-contaminating or graphene-modifying elements like chemical composition, binding partners, or oxidation state, XPS is the method of choice.

### Characterization of Graphene Dispersions

**Figure 5:**
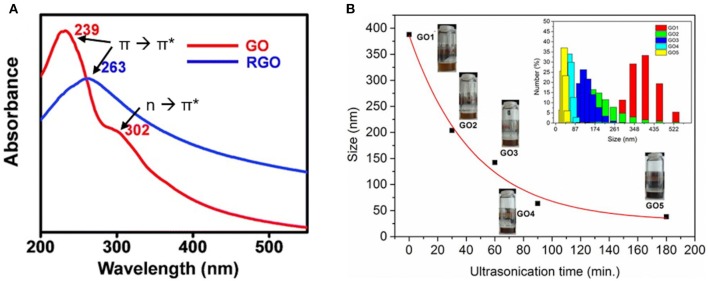
**(A)** The UV–vis spectra of GO and rGO with corresponding shift of π➔π* and reduction of *n*➔π*. **(B)** The DLS measurements of different GO flake sizes with corresponding sonication time. **(A)** Reproduced and adapted under a Creative Commons Attribution (CC BY 3.0) license from Mohandoss (2017). **(B)** Reproduced under a Creative Commons Attribution (CC BY NC-ND 4.0) license from Gonçalves et al. (2015).

The transfer of top-down synthesized graphene is preferably done by dispensing, spin-coating, or inkjet printing on the desired target as these are fast and controlled processes which can be easily applied in any mass fabrication process (Torrisi et al., 2012; Kymakis et al., 2013). For that, the graphene flakes need to be suspended in a liquid that provides high colloidal stability. Therefore, the graphene flake suspensions are often stabilized by substances like sodium cholate (Lotya et al., 2010), ethyl cellulose, or terpineol (Secor et al., 2014), which match the surface energy of the nanomaterial. The resulting graphene inks need to be analyzed in terms of concentration or stability. A big advantage of characterization directly in solution is that the chemical environment of the 2D material needs not to be changed. Measuring the extinction at a wavelength of around 230 nm reveals the concentration of the graphene inks (Wang et al., 2009). Since the extinction of the graphene oxide suspensions is very high, dilution is necessary, which might be an issue in the case of the composite materials consisting of one material of low-absorption coefficient. Furthermore, by using UV–vis, it is possible to get a first impression on the chemical structure of the graphene oxide. By determining λ_max_, typically in the range of around 230 nm, information about the amount of sp^2^ hybridization can be obtained. A redshift of λ_max_ is attributed to more π➔π^*^ transitions which are equivalent to a more ordered structure and larger sp^2^ domains (Figure 5A). A shoulder appearing at a wavelength of around 300 nm indicates the *n*➔π^*^ transition of carbonyl groups (Marcano et al., 2010).

It would also be a nice feature to get information on the flake size directly from the dispersion as it was performed by DLS (Figure 5B) (Gonçalves et al., 2015; Kumar et al., 2019). Nevertheless, DLS is measuring the hydrodynamic radius and not the absolute graphene flake size. Furthermore, the method of DLS is usually based on spherical particles, and therefore data analysis has to be performed carefully for the 2D materials (Liscio et al., 2017).

The fabrication methods based on chemical exfoliation of graphite to graphene suffer from contaminations of sulfur or sodium which are introduced during synthesis and which can only be eliminated by excessive dialysis protocols. The determination of these contaminations directly in suspension is very important since the impurities have an influence on the performance as well as on the lifetime of a graphene application (Mazánek et al., 2018). A very practical method to detect contaminations in a liquid environment is inductively coupled plasma (ICP) spectrometry. By ICP—regardless if ICP-MS or ICP-AES—qualitative information about contaminations down to the ppb/ppt range can be identified directly in the suspensions without any dilution, and therefore no change in the chemical environment has to be taken into account. To avoid or decrease the contaminations, knowledge about the origin of the impurities is very important. Therefore, the contaminations of several graphene oxide syntheses were investigated and compared by ICP-MS to prove that the kind of impurities depends on the chemicals used during synthesis (Chua et al., 2014). Higher amounts of potassium and manganese were found in GO suspensions which were fabricated by Hummer's method compared to those of other GO syntheses like those of Staudenmaier or Hofmann (Wang et al., 2014). Also, the metallic impurities can be detected. This is very helpful to characterize the purification process of GO syntheses (Barbolina et al., 2016). Despite the contaminations, modifications can also be characterized by ICP. Gao et al. determined the cobalt content (35.8%) of their Co/rGO composite by ICP-OES (Gao et al., 2019).

Nevertheless, to get more detailed information like oxidation state or binding partners, XPS measurements are inescapable. UV–vis and DLS are not ideal methods to characterize graphene in terms of concentration (high absorbance) and flake size, but both methods can be performed very easily and are cheap and fast; therefore, they must not be neglected for fast screening. Due to this, both methods can be used to check the graphene concentrations in inks or to check their stability in terms of agglomeration. In the case of the graphene quantum dots (GQDs), these are characterized as nanometer-sized fragments of graphene that show unique properties especially in their luminescence, making these materials attractive for bio-applications (Bacon et al., 2014). Photoluminescence (PL) properties are useful for optoelectronic applications (Eda et al., 2010; Wang et al., 2015). Whereas pristine graphene is characterized by a zero band gap (Li et al., 2012) and therefore no PL can be observed, GQDs' dispersions are well-known for band gap and their luminescence when they are excited by a specific wavelength (Coleman et al., 2017). Many parameters, e.g., flake size, shape, functionalities, or pH value, influence the GQDs' band gap and therefore the PL properties (Wang et al., 2015). Since the electron hole recombination at the newly formed sp^2^ domains of the rGO exhibits blue fluorescence, the PL of rGO (around 450 nm) is blue-shifted compared to the PL of GO (around 600 nm) (Chien et al., 2012). Furthermore, the PL is also strongly influenced by the flake size. For flakes that are too large, when the bandgap becomes zero and no PL can be monitored any longer, this material is used as a quencher in bioanalytical applications. The quenching of the luminescence of a dye can also be used for the characterization of the carbon nanomaterial, e.g., to evaluate the successful reduction of GO to rGO. A red shift in the PL can be observed when the size of the N-doped GQDs is increased (Tang et al., 2014).

## Summary

Despite the outstanding properties ascribed to the 2D nanomaterials which have been demonstrated and extensively reviewed (Cheng et al., 2017; Tan et al., 2017; Jin et al., 2018), only a few practical applications utilizing these materials have reached the market. One of the major hurdles in this field can be found in the inconsistency of the experimental details and the characterizations reported in literature. What is still missing is a standard of minimum information which needs to be reported. To improve the reproducibility and to allow the comparison of 2D materials fabricated or applied by different groups, it is suggested that parameters such as flake size, number of layers, morphology, functionalities, or colloidal properties of the graphene and graphene-related materials should be reported. It is shown by this review that, for all essential parameters listed, procedures for characterization are available in a great number. For the future, the development of new characterization techniques and the improvement and hyphenation of already existing methods are desirable, especially in terms of getting faster or higher throughput. Especially improvements in the surface sensitivity and the detection of contaminations will enable a better understanding of processing the 2D materials. A reduction of the acceleration voltage of SEM or STEM by maintaining the high resolution or an improved quantification of elements for XPS or AES would be helpful. Future techniques should be able to identify local element doping, defects, or contaminations at the atomic scale. STM, combined further with non-commonly used techniques such as excitation by external optical, magnetic, or electric fields, would be very beneficial to investigate and observe the graphene characteristic properties in atomic resolution. Completely missing until now are methods which will enable the online characterization of dynamic processes with high resolution. Such real-time studies are expected to provide new insights in material properties when applied to mechanical, electrical, or electromagnetic stress.

## Author Contributions

All authors listed have made a substantial, direct and intellectual contribution to the work, and approved it for publication.

### Conflict of Interest

The authors declare that the research was conducted in the absence of any commercial or financial relationships that could be construed as a potential conflict of interest.
